# Health care expenses impact on the disability-adjusted life years in non-communicable diseases in the European Union

**DOI:** 10.3389/fpubh.2024.1384122

**Published:** 2024-04-10

**Authors:** Margarida Torres, Alcina Nunes, João P. Martins, Pedro L. Ferreira, Rui Pimenta

**Affiliations:** ^1^Escola Superior de Saúde, Instituto Politécnico do Porto, Rua Dr. António Bernardino de Almeida, Porto, Portugal; ^2^UNIAG, Instituto Politécnico de Bragança, Bragança, Portugal; ^3^CEAUL – Centro de Estatística e Aplicações, Faculdade de Ciências, Universidade de Lisboa, Lisbon, Portugal; ^4^Centre for Health Studies and Research of University of Coimbra, Centre for Innovative Biomedicine and Biotechnology, Coimbra, Portugal; ^5^Faculty of Economics, University of Coimbra, Coimbra, Portugal

**Keywords:** health expenditure, health policy, disease burden, panel data, chronic diseases, public health expenditure, private health expenditure

## Abstract

**Background:**

Non-communicable diseases are a global health problem. The metric Disability-Adjusted Life Years was developed to measure its impact on health systems. This metric makes it possible to understand a disease’s burden, towards defining healthcare policies. This research analysed the effect of healthcare expenditures in the evolution of disability-adjusted life years for non-communicable diseases in the European Union between 2000 and 2019.

**Methods:**

Data were collected for all 27 European Union countries from Global Burden of Disease 2019, Global Health Expenditure, and EUROSTAT databases. Econometric panel data models were used to assess the impact of healthcare expenses on the disability-adjusted life years. Only models with a coefficient of determination equal to or higher than 10% were analysed.

**Results:**

There was a decrease in the non-communicable diseases with the highest disability-adjusted life years: cardiovascular diseases (−2,952 years/10^5^ inhabitants) and neoplasms (−618 years/10^5^ inhabitants). Health expenditure significantly decreased disability-adjusted life years for all analysed diseases (*p* < 0.01) unless for musculoskeletal disorders. Private health expenditure did not show a significant effect on neurological and musculoskeletal disorders (*p* > 0.05) whereas public health expenditure did not significantly influence skin and subcutaneous diseases (*p* > 0.05).

**Conclusion:**

Health expenditure have proved to be effective in the reduction of several diseases. However, some categories such as musculoskeletal and mental disorders must be a priority for health policies in the future since, despite their low mortality, they can present high morbidity and disability.

## Introduction

Burden disease is defined as the difference between a population’s current state of health and the optimal state of health, where the whole population achieves a full life without suffering a major illness (1).

There are several methodologies to quantify the burden of disease. However, to be able to compare between countries, the most used measure is the Disability Adjusted Life Years (DALY) which is equal to the sum of years of life lost due to premature death (YLL) and years lived with disability (YLD) (2–4). Thus,


(1) DALY=YLL+YLD

This work focuses on non-communicable diseases (NCD), known as chronic diseases, which tend to result from a combination of genetic, physiological, environmental, and behavioural factors (5, 6). According to the World Health Organization (WHO), their impact increased from 61% of global deaths in 2000 to 74% in 2019, causing 63% of DALYs in that year (compared to 47% in 2000). In Europe, NCD affects life expectancy and is responsible for 77% of the total disease burden (5, 7). The literature points to a more significant burden of cardiovascular diseases, neoplasms, chronic respiratory diseases (such as Chronic obstructive pulmonary disease and Asthma) and diabetes within NCD, accounting for more than 33 million deaths in 2019 (an increase of 28% compared to the year 2000), with at least 80% of all heart attacks, diabetes and strokes, and 40% of cancers could be prevented by monitoring the main risk factors - tobacco, alcohol, poor diet, physical inactivity and environmental factors (5, 6, 8).

NCDs were included in the WHO agenda for Sustainable Development 2030, with the goal of reducing the probability of death resulting from the four main diseases by one-third, for ages between 30 and 70 years, by 2030 (6). Moreover, the European Commission launched the *Healthier Together—EU Non-Communicable Diseases* initiative as a way of helping European Union (EU) countries to achieve that goal through the identification and implementation of effective policies and actions to reduce the burden of the NCD, which shows the topicality of this topic (7).

The economic consequences of NCDs significantly impact health care and decrease productivity. NCDs are the most significant cause of health expenditure (2).

Global Health Expenditure Database (GHED) is the largest international expense comparison database across almost 190 countries since its inception in 2000 (9). It includes financing source indicators such as current healthcare expenses (CHE), domestic general government health expenditures (GGHE-D), and domestic private expenditures (PVT-D), which include household out-of-pocket payments (OOP) (9). EU health systems vary in organisation and financing as their governance relies mainly on national legislation. However, ensuring universal access and delivering high-quality care at an affordable price for all citizens are recognised as essential societal needs as they are fundamental values and principles within the EU (10).

Therefore, the growing population ageing and the subsequent rise in demand for healthcare services present a significant challenge to the health economy (11, 12). Healthcare expenditures are a significant part of the national budgets of the EU countries. In 2020, it was equivalent to approximately 11% of the gross domestic product (13, 14). As disability becomes a large component of disease burden, it represents a high component of health expenditure and, in addition, there is also a loss of productivity and labour (7, 11).

It becomes crucial to anticipate trends and formulate adequate policies (11, 12). Thus, policymakers need to recognise the significance of DALYs as they reflect the disease burden that healthcare systems must effectively address. This highlights the importance of assessing the effect of these expenses in improving the health of EU citizens (15, 16). Healthcare expenditure is not the sole determinant of health outcomes such as DALYs. However, it plays a significant role in the accessibility, quality, and effectiveness of healthcare services, all of which ultimately influence population health outcomes. Thus, this research has two aims: to analyse the evolution of DALYs in NCDs and the health expenditures in the EU, and to evaluate the effect of health expenditures on the evolution of DALYs in NCDs.

## Methods

### Databases and variables

A multinational retrospective longitudinal study was performed. Data were collected for all 27 EU countries for the period 2000 to 2019, from 3 databases:

- Global Burden of Disease (GBD) 2019 for YLL and YLD and therefore for DALY, as described by Equation 1, related to communicable, maternal, neonatal and nutritional diseases (CMND), injuries (INJU), NCD and each NCD;
- GHED for health expenditure data;
- EUROSTAT database for population data (17).

Detailed descriptions of the health expenditure variables can be found in Supplementary Table S1.

The collected YLL, YLD and DALY values were adjusted for a standardised age and for both sexes.

### Statistical analysis

Data treatment was performed using STATA^®^ (version 14.2) and Microsoft Excel^®^ 365. First, an exploratory data analysis was carried out which included a weighted average of DALY and YLD, and some graphical representations. For a better analysis of expenditures within the private sector, private expenditure ($E_{\mathrm{Prv}}$) was generated by the difference between PVT-D and the out-of-pocket expenditure ($E_{OOP}$), and, for uniformity in the reading of the results, a logarithmization of the DALYs referring to each NCD was carried out in STATA^®^.

Secondly, a econometric panel data models were used to assess the impact of healthcare expenses on DALYs, through cross-sections (analysis of between countries in a given year) and chronological sequences (analysis of a country over the years). To avoid collinearity issues, the analysis was performed in two steps: the first step consisted in the analysis of fixed effects (FE) and random effects (RE) models for the DALYs of a NCD for country i at time t ($DALY_{it}$) with the total of health expenditures ($E_{\mathrm{Tot}}$) as the single covariate. The FE model can be described by Equation 2,


(2) $$DALY_{it}=\beta_{0}+\beta_{1}\times E_{{\mathrm{Tot}}_{it}}+\mu_{i}+\varepsilon_{it}$$

Where $\beta_{0}$ is a constant, $\beta_{1}$ is the coefficient of the independent variable, $\mu_{i}$ are the country-specific effects that are assumed constant over time and verify $\underset{i}{\sum }\mu_{i}=0$, and $\varepsilon_{it}$ are the normal error terms. The RE model is given by Equation 3,


(3) $$DALY_{it}=\beta_{0}+\beta_{1}\times E_{{\mathrm{Tot}}_{it}}+\alpha_{i}+\varepsilon_{it}$$

Where $\alpha_{i}$ stand for the country-specific effects that are now assumed to be normal random variables with null mean and equal variance, and $\epsilon_{it}$ are the normal error terms.

The second step was to consider as explanatory variables all possible combinations between $E_{\mathrm{Pub}}$, $E_{\mathrm{Prv}}$ and $E_{OOP}$. Both FE and RE models were considered.

The option between the FE and RE models was based on the result of the Hausman test for a significance level of 5%. The BIC (Bayesian information criterion) was also used to obtain a parsimonious selection of independent variables (18–21). Only models with an overall coefficient of determination equal to or higher than 10% were analysed (22).

## Results

### Evolution of DALYs

**Figure 1:**
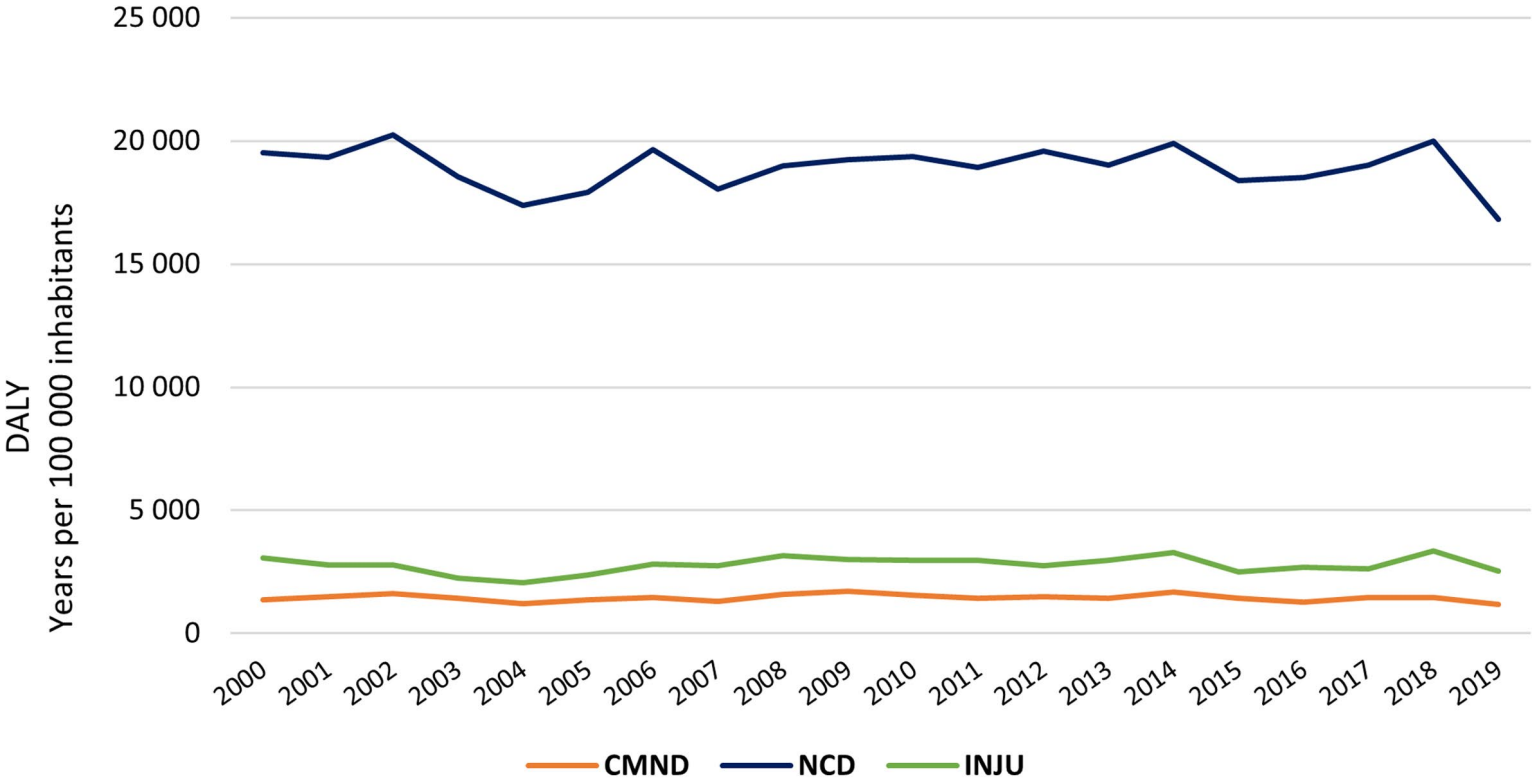
Evolution of DALYs for DNC, CMND and INJ in the EU from 2000 to 2019; CMND, communicable, maternal, neonatal and nutritional diseases; NCD, non-communicable diseases; INJU, injuries.

The evolution of DALYs for NCD, CMND and INJ in the EU from 2000 to 2019 is detailed in Figure 1. The burden of NCDs is significantly higher than the burden of CMND or INJU since the minimum for NCDs (16,800 years per $10^{5}$ inhabitants in 2019) is more than five times greater than the maximum number of injuries (3350.28 years per $10^{5}$ inhabitants in 2018) and about ten times greater than the CMND maximum (1719 years per $10^{5}$ inhabitants in 2009).

**Figure 2:**
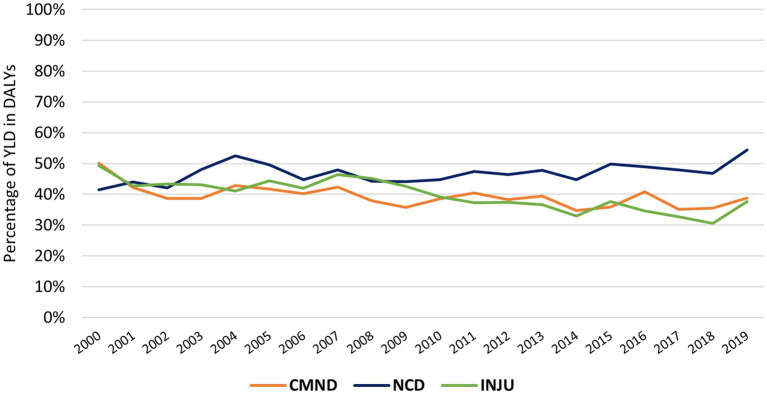
Evolution of the YLD percentage in DALYs for DNC, CMND and INJ in the EU from 2000 to 2019; CMND, communicable, maternal, neonatal and nutritional diseases; NCD, non-communicable diseases; INJU, injuries.

Despite the tendency to maintain DALYs, the percentage of these due to YLD has changed. While the CMND and INJU had a decrease in the rate of YLD within the DALYs (50.14 to 38.85% and 49.28 to 37.64%, respectively), the NCDs show an increase in the burden of YLD within DALYs, rising from 41.45 to 54.46% (Figure 2).

**Figure 3:**
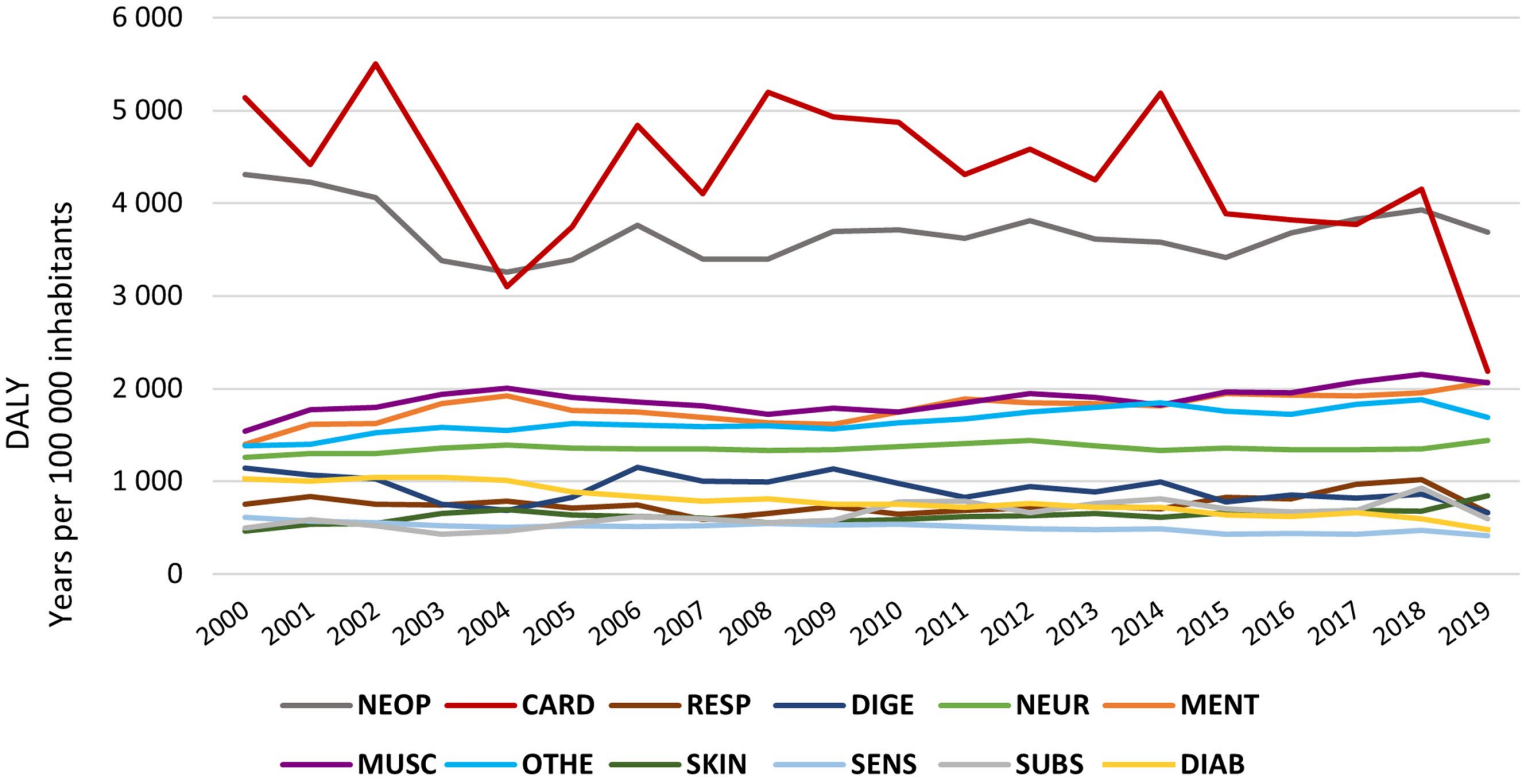
DALY’s evolution by NCD in the EU from 2000 to 2019; NEO- neoplasm; CARD, cardiovascular disease; Resp., chronic respiratory disease; DIGE, digestive disease; NEUR, neurological disorders; MENT, mental disorders; MUSC, musculoskeletal disorders; OTHE, other non-communicable disease; SKIN, skin and subcutaneous disease; SENS, sense organ disease; SUBS, substance use disorders; DIAB, diabetes and kidney disease.

When analysing the evolution of DALYs within NCDs (Figure 3), cardiovascular diseases presented the highest DALY values within NCDs. During the period under review, these diseases had a positive evolution with a decrease over time (maximum 5,502 years per $10^{5}$ inhabitants in 2002, and minimum 2,189 years per $10^{5}$ inhabitants in 2019).

Neoplasms were the second most impactful NCD, with recorded values exceeding 4,000 years per $10^{5}$ inhabitants during the initial three years under analysis, and in subsequent years these values consistently remained below that threshold.

Musculoskeletal and mental disorders and other NCDs change their position in terms of rank over time. However, all showed increasing values of DALYs. In 2019, mental disorders were the third NCD, followed by musculoskeletal disorders and other NCDs.

Neurological disorders is the sixth NCD with the most significant effect, with the lowest values in the first three years under analysis. The two highest records are found in the last decade (1,443 years per $10^{5}$ inhabitants in 2012 and 1,438 years per $10^{5}$ inhabitants in 2019), which indicates an upward trend.

**Figure 4:**
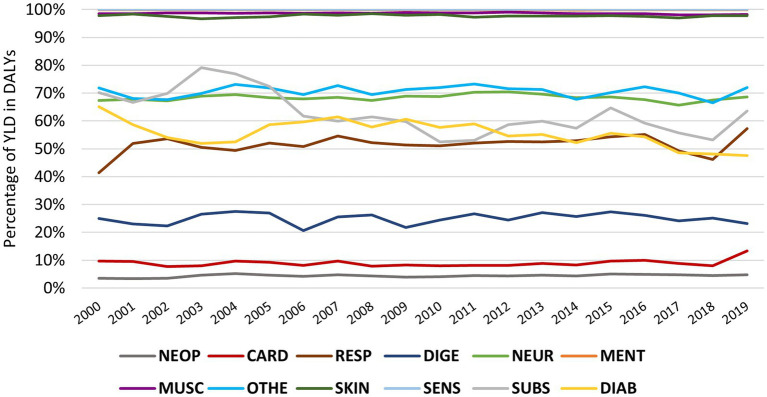
Evolution of YLD within DALYs by NCD in the EU from 2000 to 2019; NEO, neoplasm; CARD, cardiovascular disease; Respiratory chronic respiratory disease; DIGE, digestive disease; NEUR, neurological disorders; MENT, mental disorders; MUSC, musculoskeletal disorders; OTHE, other non-communicable disease; SKIN, skin and subcutaneous disease; SENS, sense organ disease; SUBS, substance use disorders; DIAB, diabetes and kidney disease.

Conversely, there has been a notable downward trend in the percentage of YLD (Figure 4) in diabetes and kidney diseases (maximum of 65.10% in 2000 and minimum 47.58% in 2019) and substance use disorders (maximum of 79.12% in 2003 and minimum 52.41% in 2010), while chronic respiratory diseases follow an increase in the percentage of YLD (minimum 41.43% in 2000 and maximum 57.22% in 2019). When analysing the variation in the rate of YLD between 2000 and 2019, these same diseases were the only ones with changes exceeding five percentual points.

### Health expenditures

**Figure 5:**
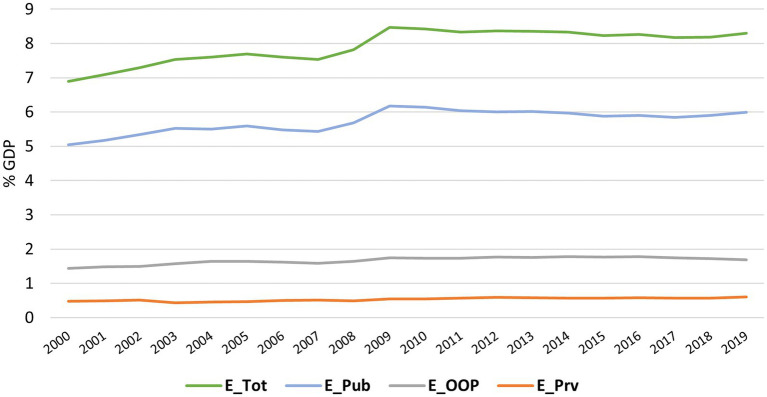
Evolution of Health Expenditure in the EU from 2000 to 2019; E_Tot, Total Health Expenditure; E_Pub, Public expenditure on health; E_Prv, Private health expenditure without out-of-pocket payments; E_OOP, Private health expenditure in the form of out-of-pocket payment.

The total of health expenditures ($E_{\mathrm{Tot}}$), public and private, has increased since 2000 (6.90% of GDP), reaching the maximum value in 2009 (8.47% of GDP) and remaining above 8.00% until the end of the study period (cf. Figure 5). The maximum expenditure occurred in 2019 in Germany (11.70% of GDP), while the minimum was in 2000 in Romania (4.21% of GDP).

**Figure 6:**
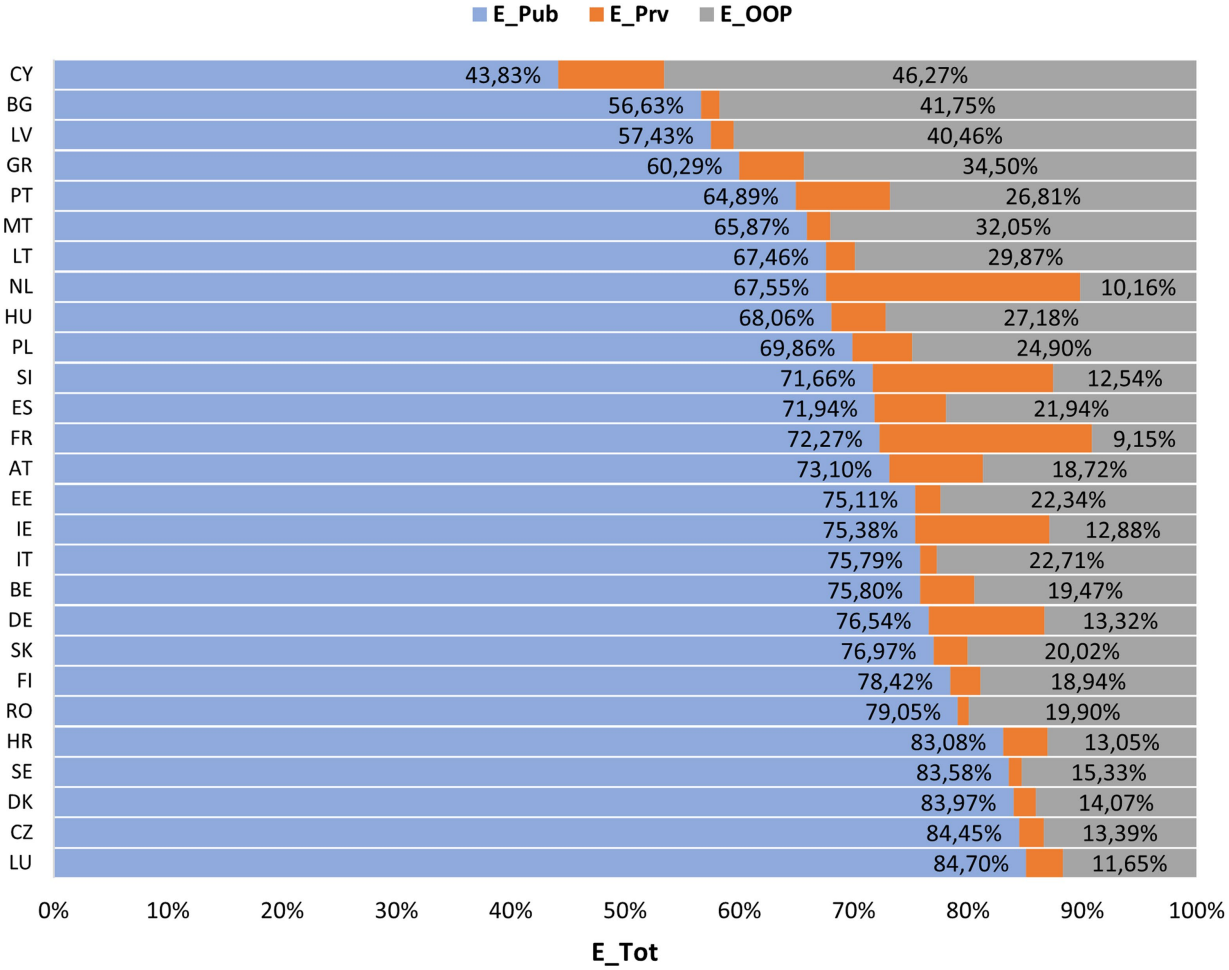
Percentage ratio of public, private and OOP expenditures within total health expenditures by country. E_Pub- Public expenditure on health; E_Prv, Private health expenditure without out-of-pocket payments; E_OOP, Private health expenditure in the form of out-of-pocket payments; E_Tot, Total health expenditure; Germany (DE), Austria (AT), Belgium (BE), Bulgaria (BG), Czechia (CZ), Cyprus (CY), Croatia (HR), Denmark (DK), Spain (ES), Slovakia (SK), Slovenia (SI), Estonia (EE), Finland (FI), France (FR), Greece (GR), Hungary (HU), Ireland (IE), Italy (IT), Latvia (LV), Lithuania (LT), Luxembourg (LU), Malta (MT), Netherlands (NL), Poland (PL), Portugal (PT), Romania (RO) and Sweden (SE).

Public expenditure ($E_{\mathrm{Pub}}$) was the $E_{\mathrm{Tot}}$ component with the most significant impact on health, its evolution over time was similar to the total growth. Thus, $E_{\mathrm{Pub}}$ attained its minimum in 2000 (5.04% of GDP) and its maximum in 2009 (6.18% of GDP), maintaining approximately 6.00% of expenditure afterwards. When examining the data by country (Figure 6), significant variations in values were observed. Sweden, the country with the highest $E_{\mathrm{Pub}}$, presented an expenditure of 9.28% of GDP in 2018 which is three times higher than Cyprus in the same period (2.88% of GDP in 2018).

On the other hand, private sector expenditures (Private expenditure ($E_{\mathrm{Prv}}$) and out-of-pocket expenditure ($E_{\mathrm{OOP}}$)), had a similar evolution over time. On average, both types of expenditure reached their minimum recorded at the beginning of the period under study, although the maximum of $E_{\mathrm{Prv}}$ occurred in 2019 (0.60% of GDP) and of $E_{\mathrm{OOP}}$ in 2014 (1.78% of GDP). By country, the Netherlands had the highest $E_{\mathrm{Prv}}$ in 2017 (2.49% of GDP), while for $E_{\mathrm{OOP}}$, Bulgaria had the highest in 2012 (3.60% of GDP). The countries with the lowest $E_{\mathrm{Prv}}$ and $E_{\mathrm{OOP}}$ were, respectively, Slovakia in 2004 (0.003% of GDP) and Luxembourg in 2019 (0.52% of GDP).

In short, all health expenditures have an upward trend with stabilisation in the last decade. By analysing the typology of health expenditure (Figure 6), it is possible to observe that Cyprus was the only EU country with an $E_{\mathrm{Pub}}$ mean lower than 50% of $E_{\mathrm{Tot}}$ (being 46.27% of these expenditures by $E_{\mathrm{OOP}}$), followed by Bulgaria and Latvia with ana $E_{\mathrm{OOP}}$ higher than 40%. Most countries have an $E_{\mathrm{Prv}}$ and $E_{\mathrm{OOP}}$ lower than 30% (17 countries), thus there is a higher expenditure on health by public agencies. $E_{\mathrm{OOP}}$ represent more than 50% of non-public expenditure in most countries, with a median of almost 20% of $E_{\mathrm{Tot}}$. France (9.15%), Netherlands (10.16%) and Luxembourg (11.65%) have the lowest percentages of $E_{\mathrm{OOP}}$.

### Panel data models

The panel data models were found to model the evolution of the DALYs of each NCD with $E_{\mathrm{Tot}}$, as the only explanatory variables are detailed in Supplementary Table S2. Four of the NCDs presented a low overall *r*^2^ (chronic respiratory disease, substance use disorders, diabetes and kidney disease and other non-communicable disease). Mental disorders despite an overall *r*^2^ of more than 30%, did not present statistically significant for $E_{\mathrm{Tot}}$. As for the other NCDs, all had a significant favourable evolution, except for musculoskeletal disorders, where, according to the fitted model, a 1% increase in $E_{\mathrm{Tot}}$ increased DALYs by 0.26%. The evolution was especially favourable in cardiovascular and digestive diseases.

To determine the effect of the expenditures $E_{\mathrm{Pub}}$, $E_{\mathrm{Prv}}$ and $E_{\mathrm{OOP}}$ on the evolution of the DALYs of each NCD, panel data models were fitted for every combination of the explanatory variables. The most parsimonious model, according to the BIC criteria, was chosen and its parameters are presented in Table 1 (results for diseases with an overall *r*^2^ less than 10% were omitted). Supplementary Table S3 shows the complete results.

**Table 1:** Most parsimonious panel data model for NCD DALYs by $E_{\mathrm{Pub}}$, $E_{\mathrm{Prv}}$, $E_{\mathrm{OOP}}$.

	NEO	CARD	DIGE	NEUR	MUSC	SKIN	SENS
PDM	RE*	FE*	RE*	FE*	FE*	FE*	FE*
$E_{Pub}{\;}^{a}$	−2.99%* (−4.03,-1.95)	−10.37%* (−12.70,-8.05)	−4.35%* (−6.00,-2.69)	−0.34%* (−0.51,-0.16)	0.32%* (0.19,0.45)		−0.33%* (−0.47,0.20)
$E_{Prv}{\;}^{a}$	−7.14%* (−10.84,3.44)	−45.32%* (−53.75,-36.88)	−23.05%* (−29.01,-17.09)			−0.70%* (−1.01,-0.39)	−1.90%* (−2.40,1.41)
$E_{OOP}{\;}^{a}$	−6.25%* (−9.10,3.40)	−18.54%* (−25.14,.-11.94)	−7.08%* (−11.70,-2.45)			−0.59%* (−0.82,-0.36)	−0.95%* (−1.34,-0.56)
Overall $r^{2}$	13.83%	46.82%	28.32%	18.05%	25.52%	13.31%	29.10%

PDM, Panel data model; $E_{Pub}$, Public expenditure on health; $E_{Prv}$, Private health expenditure without out-of-pocket payments; $E_{OOP},$ Private health expenditure in the form of out-of-pocket payments; RE, random effects model; FE, fixed effects model; NEO, neoplasm; CARD, Cardiovascular disease; DIGE, digestive disease; NEUR, neurological disorders; MUSC, Musculoskeletal disorders; SKIN, skin and subcutaneous disease; SENS, Sense organ disease; *Significant at a 1% level; ^a^coefficient obtained in the most parsimonious panel data model according to the BIC criterion; 95% confidence intervals are presented in brackets.

Public health expenditure has a significant effect on all NCDs except for skin and subcutaneous diseases. However, in musculoskeletal disorders, the increase in public expenditure does not have a positive impact on DALYs (a 1% increase in $E_{\mathrm{Pub}}$ increases its DALYs by 0.32%).

Upon analysing both the private and public sector expenses, it becomes clear that these expenses have a significant impact on NCDs, with cardiovascular diseases showing the coefficients with the greatest improvements for $E_{\mathrm{Pub}}$ (10.37%), $E_{\mathrm{Prv}}$ (45.32%) and $E_{\mathrm{OOP}}$ (18.54%). Comparing the types of expenditure, the impact of the increase in private sector expenditure ($E_{\mathrm{Prv}}$ and $E_{\mathrm{OOP}}$), in percentage terms, always showed an expected variation greater than that of $E_{\mathrm{Pub}}$, in all models.

## Discussion

### Evolution of DALYs

In this study, we assessed trends in DALYs and health expenditure across the 27 EU countries over 20 years (2000–2019), and analysed the effect of health expenditure in the DALYs evolution.

According to GBD 2019 Diseases and Injuries Collaborators (2020), with the increase in the sociodemographic index, there is an inversion of the burden from CMND to NCDs, where the contribution of YLD to DALYs becomes greater. This research also observed that NCDs present a more significant burden for health systems compared to CMND and injuries, as well as the trend of increasing disability, measured by YLD, for these diseases.

Despite the higher burden of NCDs, there was a maintenance of DALYs in the EU over time, through a downward trend in diseases such as cardiovascular and neoplasms and an upward trend for musculoskeletal and mental disorders. Daroudi et al. also observed a maintenance of DALYs for NCDs between 2000 and 2016 (worldwide), while Liu et al. observed a downward trend worldwide in DALYs for musculoskeletal disorders between 1990 and 2019 (23).

The drop of more than 1900 years per $10^{5}$ inhabitants in cardiovascular diseases in 2019 should be carefully analysed since the mean in 2018 is lower than the three lower minimum values found for the same variable over the period analysed. This fall needs further studies and analysis in subsequent years.

Regarding the contribution of the YLD in the evolution of the DALYs, the NCDs have shown different performances. DALYs concerning cardiovascular, digestive, and sensory organ diseases decreased, and the YLD percentage remained similar over time. DALYs due to diabetes and kidney disease fell, but the YLL percentage increased. On the other hand, skin and subcutaneous diseases, chronic respiratory diseases, other NCDs and neurological, musculoskeletal and mental disorders had a trajectory of increased DALYs with YLD percentage showing low variation. However, disorders due to substance use increased DALYs and decreased the YLD percentage. Thus, most NCDs maintain the percentage of DALYs components over time, except for diabetes and kidney disease and disorders due to substance use, where there is an increase in premature deaths, and chronic respiratory diseases, with an increase in disability. Moreover, in the study by GBD 2019 Diseases and Injuries Collaborators, worldwide, an increase in YLL for disorders due to substance use was observed, justified by the inadequate prescription of opiates or fentanyl abuse. On the other hand, Kotwas et al., in a study in central Europe, observed an increase in DALYs for type 2 diabetes mellitus with an increase in YLDs (24).

### Healthcare expenditures policies in the EU

EU adhere to the principle of universal access to healthcare, which is achieved through compulsory funding for the public sector, and there is no country in the EU (and very few worldwide) in which the private sector is the only source of access to health (25). Therefore, in this study, all EU countries financed their health systems through the public and private sectors, where, on average, there was an upward trend in all types of expenditure over time, with $E_{\mathrm{Pub}}$ being the ones that most contribute to $E_{\mathrm{Tot}}$, followed by $E_{\mathrm{OOP}}$ and $E_{\mathrm{Prv}}$. Note that Cyprus was the only country with a $E_{\mathrm{Pub}}$ component below 50%, as in WHO et al., being the value justified by the inability of the Cypriot health system to cover 10% of the population, motivating a significant reform in the health system in 2019 (26).

Thus, public entities were the major funders of health systems in EU countries, reaching over 80% in Croatia, Sweden, Denmark, Czechia and Luxembourg.

Figure 5 shows a sudden increase in health expenditure in 2009. This increase, as described by OECD & European Commission (2020), is due to a contraction in GDP due to the 2008 financial crisis and not to increased funding for health.

Both this study and WHO et al. observed a higher burden with $E_{\mathrm{OOP}}\;$compared to $E_{\mathrm{Prv}}\;$, except in France, Slovenia and the Netherlands. These results may be due to its quick access, the provider’s choice or better facilities provided by the private system. However, it also shows a deficit in the articulation of health subsystems and the private sector, since $E_{\mathrm{OOP}}$ are borne directly by users, withdrawing income or savings from households. In the EU, on average, 1/5 of total health expenditure is paid out-of-pocket, mainly for pharmaceutical, dental and other long-term healthcare services (26).

### The effect of health expenditures on DALYs

The NCDs received, until 2019, a residual investment, mainly compared to the expenses in diseases such as AIDS, tuberculosis, malaria and neonatal, child and maternal health (27). However, it is estimated that a 1% increase in per capita health expenditure reduces DALYs for all causes by 0.24%, and in countries with a high development index [as in the 27 EU countries (28)] the decrease in DALYs reaches 0.27% (29). This highlights the importance of analysing the impact of the health expenditure (30) in the DALYs for each category of disease as its potential benefit has been previously reported in other studies (31, 32). Our findings suggest that benefits regarding neoplasms, cardiovascular and digestive diseases are significantly higher than the estimated benefit of 0.27%, for all causes, estimated by Daroudi et al.

Most health expenditures are related to public organisations, reflecting fewer changes in DALYs compared to the private sector. All increases in $E_{\mathrm{Prv}}$ and/or $E_{\mathrm{OOP}}$ decrease the DALYs of NCDs, while the increase in $E_{\mathrm{Pub}}$ always shows less significant improvements compared to the private sector or even the increase in DALYs (as for musculoskeletal disorders). On the other hand, despite $E_{\mathrm{OOP}}$, in most countries, being responsible for more than 50% of expenditures in the private sector, the health outcomes for increasing these expenditures are always lower than the results with increasing $E_{\mathrm{Prv}}$.

Neurological disorders increased DALYs over time, however, only $E_{\mathrm{Pub}}$ significantly contributed to the decrease in their impact. Thus, a deeper private sector involvement should be considered in the future. Conversely, skin and subcutaneous diseases presented an increase in their DALYs and its evolution was only influenced by the private sector. A bigger contribution from the public sector would be important to face this increase.

Cardiovascular diseases showed the most significant effect on health systems through DALYs and simultaneously had the most favourable evolution when there was a 1% increase - in any health expenditure type - translating into a decrease in DALYs between 10 and 45%! These results show considerable attention to this pathology, justifying the downward DALY trend over time. Otherwise, musculoskeletal disorders showed the worst increasing trends for the period under study. As for the musculoskeletal, it was the only NCD in which the increase in $E_{\mathrm{Tot}}\;$and/or $E_{\mathrm{Pub}}$ did not show positive results in the health of the population. Mental diseases showed a poor relation between DALYs and expenditures (overall $r^{2}$ equal to 1%). This result shows that investment, mainly public, is not responding to the needs of the population since, as advocated by GBD 2019 Diseases and Injuries Collaborators (2020), there is little development of strategies for these diseases, given the low mortality (main focus of health policies at a global level). Singh et al., in southeast Asia, found better results with public expenditure, compared to private expenditure: a 1% increase in public expenditure reduced NCD mortality by 0.6%, while private expenditure increased mortality by 0.15% (33).

Several studies have already addressed the problem of assessing the impact of healthcare expenditure on health outcomes (e.g., mortality rate) (15, 16, 22, 33). However, to our knowledge, this is the first study in which the health outcome of interest are DALYs and it is important to conduct further research in this area.

### Limitations

This study had some limitations. Firstly, the study was designed as a second analysis of GBD data, and its limitations have already been published (such as the availability of primary data and the case definition or measurement method). Secondly, only the main categories of diseases within the NCDs were analysed without considering each disease, which may bias the results. Thirdly, only health expenditures were analysed as contributing to the DALYs, and the literature points to a multifactorial impact [risk factors such as poor diet, obesity and high blood (33, 34); socioeconomic and demographic structure of the populations and health inequalities (12)]. This is particularly clear in the cases where models have a very low overall coefficient of determination *r*^2^ (e.g., mental disorders). Finally, the study did not consider the typology of health systems within the EU, relying only on definitions of health expenditure.

## Conclusion

The strategic plans implemented in cardiovascular diseases and neoplasms have yielded positive outcomes since the funding invested is associated with a more significant reduction in DALYs, with repercussions in improving the population’s health over time. Conversely, musculoskeletal must be a priority for health policies in the future since, despite their low mortality, they present high morbidity and disability, associated with an increasing evolution over time have a significant economic impact on society.

## Data availability statement

The original contributions presented in the study are included in the article/Supplementary material, further inquiries can be directed to the corresponding author.

## Author contributions

MT: Writing – original draft. AN: Writing – review & editing. JM: Writing – review & editing. PF: Writing – review & editing. RP: Writing – review & editing.
