# Supplementary material for: Health care expenses impact on the disability-adjusted life years in non-communicable diseases in the European Union
Source: Front Public Health. 2024 Apr 10;12:1384122. doi: 10.3389/fpubh.2024.1384122 (PMC11041633; doi:10.3389/fpubh.2024.1384122)
Supplement: Supplementary file 1 [file Table_1.docx]

**Table S1.** Characterization of the health expenditure variables (GBD- Global Burden of Disease; GHED- Global Health Expenditure Database)

| **Variable** | **Name** | **Source** |
| --- | --- | --- |
| ${DALY}_{n}$ | Disability-adjusted life year for cause $x$ per 100 000 inhabitants. | GBD 2019 (36) |
| $\mathrm{YLL}_{n}$ | Years of life lost due to premature mortality for cause $x$ per 100 000 inhabitants. | GBD 2019 (36) |
| ${YLD}_{n}$ | Years lived with a disability for cause $x$ per 100 000 inhabitants | GBD 2019 (36) |
| $E_{Tot}$ | Total health expenditure as a percentage of GDP | GHED (37) |
| $E_{Pub}$ | Public expenditure on health as a percentage of GDP | GHED (37) |
| $E_{Prv}$ | Private health expenditure without out-of-pocket payments as a percentage of GDP | GHED (37) |
| $E_{OOP}$ | Private health expenditure in the form of out-of-pocket payments as a percentage of GDP | GHED (37) |
| $PVT-D$ | Domestic private expenditures as a percentage of GDP | GHED (37) |
