# Supplementary material for: Health care expenses impact on the disability-adjusted life years in non-communicable diseases in the European Union
Source: Front Public Health. 2024 Apr 10;12:1384122. doi: 10.3389/fpubh.2024.1384122 (PMC11041633; doi:10.3389/fpubh.2024.1384122)
Supplement: Supplementary file 2 [file Table_2.docx]

**Table S2.** Panel data model for DNC DALYs by $E_{Tot}$. PDM- Panel data model; $E_{Tot}$- Total Health Expenditure; RE- random effects model; FE- fixed effects model; NEO- neoplasm; CARD- Cardiovascular disease; RESP- Chronic respiratory disease; DIGE- digestive disease; NEUR- neurological disorders; MENT- Mental disorders; MUSC- Musculoskeletal disorders; OTHE- Other non-communicable disease; SKIN- skin and subcutaneous disease; SENS- Sense organ disease; SUBS- Substance use disorders; DIAB- Diabetes and kidney disease **- Significant at a 1% level; *- Significant at a 5% level; 95% confidence intervals are presented in brackets.

|  | NEO | CARD | RESP | DIGE | NEUR | MENT | MUSC | OTHE | SKIN | SENS | SUBS | DIAB |
| --- | --- | --- | --- | --- | --- | --- | --- | --- | --- | --- | --- | --- |
| PDM | RE** | FE** | FE** | FE** | FE** | FE | FE** | FE** | FE** | FE** | RE | RE* |
| $\boldsymbol{E}_{\boldsymbol{Tot}}$ | -3.96% **  (-4.74,  -3.19) | -14.90%**  (-16.7,  -13.1) | -4.36%**  (-5.25,  -3.48) | -6.28%**  (-7.54,  -5.01) | -0.26%**  (-0.40,  0.12) | 0.01%  (-0.23,  0.24) | 0.26%**  (0.16,  0.36) | -4.17%**  (-4.87,  -3.48) | -0.12%**  (-0.18,  -0.06) | -0.58%**  (-0.69,  -0.48) | -0.12%  (-0.89,  0.65) | -1.01%**  (-1.74,  -0.28) |
| Overall $\boldsymbol{r}^{\boldsymbol{2}}$ | 13.77% | 53.89% | 7.28% | 29.65% | 20.32% | 32.21% | 33.48% | 0.91% | 42.50% | 41.97% | 1.02% | 8.83% |
