# Supplementary material for: Health care expenses impact on the disability-adjusted life years in non-communicable diseases in the European Union
Source: Front Public Health. 2024 Apr 10;12:1384122. doi: 10.3389/fpubh.2024.1384122 (PMC11041633; doi:10.3389/fpubh.2024.1384122)
Supplement: Supplementary file 3 [file Table_3.docx]

**Table S3**. Panel data models for NCD DALYs by $E_{\mathrm{Pub}}$, $E_{\mathrm{Prv}}$, $E_{\mathrm{OOP}}$ (best model for each NCD is highlighted). PDM- Panel data model; $\mathbf{E}_{\mathbf{Pub}}$- Public expenditure on health; $\mathbf{E}_{\mathbf{Prv}}$ - Private health expenditure without out-of-pocket payments; $\mathbf{E}_{\mathbf{OOP}}$- Private health expenditure in the form of out-of-pocket payments; RE- random effects model; FE- fixed effects model; NEO- neoplasm; CARD- Cardiovascular disease; RESP- Chronic respiratory disease; DIGE- digestive disease; NEUR- neurological disorders; MENT- Mental disorders; MUSC- Musculoskeletal disorders; OTHE- Other non-communicable disease; SKIN- skin and subcutaneous disease; SENS- Sense organ disease; SUBS- Substance use disorders; DIAB- Diabetes and kidney disease; ***- Significant at a 1% level; **- Significant at a 5% level; *- Significant at a 10% level.

|  |  | **NEO** | **CARD** | **RESP** | **DIGE** | **NEUR** | **MENT** | **MUSC** | **OTHE** | **SKIN** | **SENS** | **SUBS** | **DIAB** |
| --- | --- | --- | --- | --- | --- | --- | --- | --- | --- | --- | --- | --- | --- |
| **Model 1** | PDM | RE*** | FE*** | FE*** | RE*** | FE** | FE | FE*** | FE*** | FE*** | FE*** | RE | FE*** |
|  | $E_{Pub}$ | -2.99%*** | -10.37%*** | -2.55%*** | -4.35%*** | -0.33%*** | 0.31%* | 0.26%*** | -3.67%*** | 0.07% | -0.33%*** | 0.52% | 0.54% |
|  | $E_{Priv}$ | -7.14%*** | -45.32%*** | -11.72%*** | -23.05%*** | 0.05% | -0.35% | -0.45%* | -10.65%*** | -0.72%*** | -1.90%*** | -4.29%** | -6.18%*** |
|  | $E_{OOP}$ | -6.25%*** | -18.54%*** | -8.96%*** | -7.08%*** | -0.07% | -0.87%* | 0.53%* | -3.07%** | -0.66%*** | -0.95%*** | -0.64% | -4.92%*** |
|  | BIC | -1372.01 | -570.22 | -1295.85 | -905.11 | -3244.01 | -2706.41 | -3583.36 | -1527.05 | -4121.88 | -3615.28 | -1406.86 | -1471.25 |
|  | Overall $r^{2}$ | 13.83% | 46.82% | 6.04% | 28.32% | 17.47% | 0.10% | 14.40% | 1.06% | 5.54% | 29.10% | 8.44% | 3.42% |
| **Model 2** | PDM | FE*** | FE*** | FE*** | RE*** | FE*** | FE | FE*** | FE*** | FE*** | FE*** | RE* | FE*** |
|  | $E_{Pub}$ | -3.75%*** | -12.72%*** | -3.68%*** | -5.17%*** | -0.34%*** | 0.20% | 0.33%*** | -4.05%*** | -0.01% | -0.45%*** | 0.44% | -0.08% |
|  | $E_{Priv}$ | -9.50%*** | -49.13%*** | -13.56%*** | -24.28%*** | 0.03% | -0.53% | -0.34% | -11.28%*** | -0.86%*** | -2.10%*** | -4.40%** | -7.19%*** |
|  | BIC | -1365.22 | -545.19 | -1273.81 | -903.91 | -3250.23 | -2708.87 | -3582.54 | -1528.11 | -4098.36 | -3597.45 | -1413.09 | -1465.52 |
|  | Overall $r^{2}$ | 4.97% | 50.41% | 5.99% | 21.93% | 17.50% | 0.34% | 19.78% | 0.49% | 15.28% | 29.94% | 5.57% | 10.64% |
| **Model 3** | PDM | RE*** | FE*** | FE*** | RE*** | FE*** | FE* | FE*** | FE*** | FE*** | FE*** | RE | FE*** |
|  | $E_{Pub}$ | -3.30%*** | -11.62%*** | -2.87%*** | -5.16%*** | -0.33%*** | 0.30%* | 0.25%*** | -3.96%*** | 0.05% | -0.38%*** | 0.39% | 0.37% |
|  | $E_{OOP}$ | -6.81%*** | -24.24%*** | -10.44%*** | -9.43%*** | -0.06% | -0.92%** | 0.47%** | -4.41%*** | -0.75%*** | -1.19%*** | -1.13% | -5.69%*** |
|  | BIC | -1360.76 | -469.81 | -1272.47 | -856.82 | -3250.27 | -2712.32 | -3586.47 | -1495.95 | -4106.17 | -3564.28 | -1408.57 | -1465.91 |
|  | Overall $r^{2}$ | 19.15% | 25.52% | 2.59% | 23.91% | 18.23% | 1.03% | 30.35% | 0.66% | 0.00% | 16.77% | 4.15% | 24.67% |
| **Model 4** | PDM | RE*** | FE*** | FE*** | RE*** | RE | RE | FE*** | FE*** | FE*** | FE*** | FE | FE*** |
|  | $E_{Priv}$ | -8.80%*** | -49.08%*** | -12.65%*** | -24.99%*** | 0.03% | -0.11% | -0.36% | -11.98%*** | -0.70%*** | -2.02%*** | -3.94%** | -5.98%*** |
|  | $E_{OOP}$ | -8.67%*** | -29.13%*** | -11.56%*** | -11.05%*** | -0.43%* | -0.53% | 0.79%*** | -6.81%*** | -0.59%*** | -1.29%*** | 0.11% | -4.37%*** |
|  | BIC | -1348.08 | -500.66 | -1283.48 | -886.26 | -3238.29 | -2708.80 | -3575.89 | -1476.06 | -4125.32 | -3597.90 | -1412.41 | -1476.36 |
|  | Overall $r^{2}$ | 7.14% | 15.54% | 2.52% | 21.12% | 0.72% | 6.40% | 0.07% | 3.03% | 13.31% | 11.51% | 3.69% | 0.56% |
| **Model 5** | PDM | RE*** | FE*** | FE*** | RE*** | FE*** | FE | FE*** | FE*** | FE | FE*** | RE | FE |
|  | $E_{Pub}$ | -4.10%*** | -14.99%*** | -4.22%*** | -6.33%*** | -0.34%*** | 0.15% | 0.32%*** | -4.57%*** | -0.05% | -0.55%*** | 0.24% | -0.39% |
|  | BIC | -1357.49 | -433.13 | -1242.19 | -856.48 | -3277.40 | -2713.02 | -3597.93 | -1502.27 | -4100.43 | -3552.30 | -1425.32 | -1466.56 |
|  | Overall $r^{2}$ | 7.94% | 48.09% | 5.23% | 16.05% | 18.05% | 17.47% | 25.52% | 0.02% | 31.42% | 34.83% | 0.45% | 17.68% |
| **Model 6** | PDM | FE*** | RE*** | FE*** | RE*** | FE | FE | FE | FE*** | FE*** | FE*** | RE** | RE*** |
|  | $E_{Priv}$ | -11.97%*** | -56.26%*** | -15.98%*** | -27.79%*** | -0.19% | -0.40% | -0.13% | -13.94%*** | -0.87%*** | -2.40%*** | -4.11%** | -7.57%*** |
|  | BIC | -1320.90 | -432.94 | -1238.58 | -870.16 | -3242.05 | -2713.31 | -3564.44 | -1456.30 | -4104.55 | -3556.55 | -1418.69 | -1471.78 |
|  | Overall $r^{2}$ | 0.62% | 26.96% | 3.43% | 14.87% | 9.95% | 14.64% | 6.22% | 0.98% | 14.01% | 14.48% | 4.09% | 10.02% |
| **Model 7** | PDM | RE*** | FE*** | FE*** | RE*** | RE* | RE | RE*** | FE*** | RE*** | RE*** | RE | FE*** |
|  | $E_{OOP}$ | -9.67%*** | -36.76%*** | -13.53%*** | -14.53%*** | -0.42%* | -0.56% | 0.74%*** | -8.67%*** | -0.70%*** | -1.60%*** | -0.73% | -5.30%*** |
|  | BIC | -1332.99 | -397.48 | -1256.20 | -832.96 | -3244.53 | -2714.92 | -3580.22 | -1439.61 | -4111.02 | -3541.90 | -1414.44 | -1471.66 |
|  | Overall $r^{2}$ | 5.84% | 1.58% | 0.04% | 2.45% | 0.47% | 3.20% | 1.50% | 1.67% | 0.67% | 0.00% | 5.84% | 21.13% |
